# Allostatic load as a predictor of grey matter volume and white matter integrity in old age: The Whitehall II MRI study

**DOI:** 10.1038/s41598-018-24398-9

**Published:** 2018-04-23

**Authors:** Enikő Zsoldos, Nicola Filippini, Abda Mahmood, Clare E. Mackay, Archana Singh-Manoux, Mika Kivimäki, Mark Jenkinson, Klaus P. Ebmeier

**Affiliations:** 1 Department of Psychiatry, University of Oxford, Warneford Hospital, Oxford, OX3 7JX UK; 2 Wellcome Centre for Integrative Neuroimaging, Oxford Centre for Functional MRI of the Brain, Nuffield Department of Clinical Neurosciences, University of Oxford, John Radcliffe Hospital, Oxford, OX3 9DU UK; 3 Wellcome Centre for Integrative Neuroimaging, Oxford Centre for Human Brain Activity, University of Oxford, Warneford Hospital, Oxford, OX3 7JX UK; 4 Department of Epidemiology and Public Health, University College London, London, WC1E 7HB UK; 5 INSERM U1018, Center for Research in Epidemiology and Population Health, Villejuif, 94807 France

## Abstract

The allostatic load index quantifies the cumulative multisystem physiological response to chronic everyday stress, and includes cardiovascular, metabolic and inflammatory measures. Despite its central role in the stress response, research of the effect of allostatic load on the ageing brain has been limited. We investigated the relation of mid-life allostatic load index and multifactorial predictors of stroke (Framingham stroke risk) and diabetes (metabolic syndrome) with voxelwise structural grey and white matter brain integrity measures in the ageing Whitehall II cohort (N = 349, mean age = 69.6 (SD 5.2) years, N (male) = 281 (80.5%), mean follow-up before scan = 21.4 (SD 0.82) years). Higher levels of all three markers were significantly associated with lower grey matter density. Only higher Framingham stroke risk was significantly associated with lower white matter integrity (low fractional anisotropy and high mean diffusivity). Our findings provide some empirical support for the concept of allostatic load, linking the effect of everyday stress on the body with features of the ageing human brain.

## Introduction

Between 2015–2050 the world’s population aged over 60 will have doubled to 2 billion^1^. Perceived everyday stress^2,3^ and stress-related disorders are common^4^. The individual’s physiological stress-response to a challenging stimulus^5^ is adaptive in the short run but if it is unremitting it can accelerate ageing, promote the onset of age-related diseases, and shorten life^6^.

The allostatic load index quantifies the adverse effects of chronic stress on peripheral organ systems^7^. It summarizes the multisystem physiological response to prolonged or repeated psychological stress and includes cardiovascular, metabolic and inflammatory components at subclinical levels (hence often referred to as secondary markers; for a review, see^8,9^). Allostatic load is the conceptual basis for a comprehensive assessment of risk in the ageing process. Indexing allostatic load is linked to accelerated ageing^10^ and stress-related illnesses that are more prevalent among older adults, such as a decline in physical functioning and cognition, an increase in incident cardiovascular events and depressive symptoms^11,12^, and an increase of all-cause mortality risk^13^. The allostatic load index is a better predictor of health-related outcomes in old age than its individual components, which indicates the overall impact of physiological dysregulation across multiple systems that over time reach subclinical levels^11^.

The hippocampus, amygdala and the prefrontal cortex act as regulators, while the hypothalamic-pituitary-adrenal axis, the cardiovascular-, metabolic- and immune systems are effectors of the chronic stress response and allostatic load^9^. Despite its central role in the stress response and being a target of it^14,15^, brain research of the effect of allostatic load index is limited to particular conditions, such as schizophrenia^16,17^ and late-life depression^18^. An inverse association of the index with total brain and white matter volume, a positive (sic!) association with left hippocampal volume, and no association with global grey matter and right hippocampal volume were observed after standardizing all volumetric measures for intracranial volume to account for variations in individual brain size in the Lothian birth cohort (N = 633 (302 female), mean age = 72.5 (0.7 SD) years)^19^. More recently an inverse association of the index with grey and white matter volume and global fractional anisotropy, and no associations with white matter hyperintensity volume and global mean diffusivity were observed in the same cohort, after adjusting for sex at the age of 73 (mean age = 72.7 (0.7 SD) years, N = 657 (number of females not specified)^20^.

The Framingham stroke risk score and metabolic syndrome are multifactorial predictors of incident stroke^21^ and diabetes^22^, irrespective of experienced stress. They comprise of cardio-metabolic measures, some of which are shared with the allostatic load index. The Framingham stroke risk is linked to reduced fractional anisotropy^23^, and global and hippocampal atrophy^24^. Metabolic syndrome is associated with grey matter volume reduction in the right nucleus accumbens and global cortical thickness^25^, vascular brain changes in the form of periventricular white matter hyperintensities and subcortical white matter lesions in middle-aged individuals^26^, reduced white matter integrity in fronto-temporal regions in middle-aged and older individuals^27^ and silent brain infarction in older individuals^26^.

We investigated the relation of the allostatic load index, Framingham stroke risk and metabolic syndrome with structural grey and white matter measures. Because of the similarity in their composite scores, we were specifically interested in whether any of these markers had a unique association with brain structure, and thus were better at predicting structural brain integrity. The notion that the allostatic load index merely reflects mechanisms that are associated with metabolic syndrome in older age was previously rejected^28^. However, the three scores have not been formally compared in relation to structural brain outcomes. We used data from the Whitehall II imaging sub-study^29^, where markers were measured prospectively, decades prior to a magnetic resonance imaging scan. We hypothesized (1) that these three markers are associated with widespread reduced grey and white matter integrity after controlling for relevant socio-demographic variables. We furthermore hypothesized (2) that despite their shared variance, the three markers make a quantitatively distinct and anatomically unique contribution to grey matter volume and white matter integrity, respectively.

## Material and Methods

### Participant characteristics

The sample was drawn from the first 563 participants recruited to take part in the Whitehall II imaging sub-study between April 2012 and December 2014^29^ (https://bmcpsychiatry.biomedcentral.com/articles/10.1186/1471-244X-14-159). All participants were randomly selected from the Phase 11 examination of the Whitehall II (“Stress and Health”) study, an on-going prospective occupational cohort study conducted at University College London. The study was originally designed to explore the biological pathways through which social circumstances affect health, with a particular focus on stress that manifests as social inequality at the workplace, and cardiovascular disease and mortality outcome^30,31^. At baseline Phase 1, between 1985–1988, the Whitehall II study included 10,308 British civil service workers aged 35–55 (born between 1932–1955), of whom 6,895 were men. Follow-up health examinations were conducted over the following 30 years, approximately every five years. The collection of biological measures has been described elsewhere^32–35^. The present analysis uses data acquired at Phase 3 (1991–1994) and Phase 7 (2002–2004). Phase 11 took place between February 2012 and March 2013. A total of 74% of participants who took part in Phase 11 had reached or passed the statutory retirement age of 65. Ethical approval was obtained from the University of Oxford Central University and Medical Science Division Interdisciplinary Research Ethics Committee, and the University College London and University College London Hospital Committees on the Ethics of Human Research. All methods were performed in accordance with the relevant guidelines and regulations. All participants provided informed written consent.

### Inclusion/exclusion criteria

Participants were excluded from analysis if they did not have a magnetic resonance imaging (MRI) scan, had noticeable structural abnormalities such as strokes, or poor image quality that pre-processing and artefact correction could not fix, or had a missing secondary stress marker at either phase.

### Markers

The Framingham stroke risk score (FSRS) is a sex-specific stroke risk appraisal function that empirically relates cardiovascular risk factors to the probability of a stroke within 10 years^21^. It takes account of cardiovascular health, diabetes mellitus, smoking habits, sex and age. Metabolic syndrome (MetS) was defined based on the presence of at least three of the following five components^36^: high blood pressure or use of antihypertensives, abdominal obesity, elevated fasting glucose, high-density lipoprotein (HDL) cholesterol, and serum triglycerides (Supplementary Table S1). The cut-offs for abdominal obesity and HDL cholesterol were sex specific. Allostatic load (AL) index was defined as the linear combination of nine physiological measures with values above a high-risk threshold^37^: blood pressure, fasting glucose, fasting insulin, high- and low-density lipoprotein (LDL) cholesterol, serum triglycerides, C-reactive protein (CRP), and interleukin-6 (IL-6) (Supplementary Table S1). An elevated level of each measure carries more risk, except in case of HDL cholesterol. The sum of each score at Phase 3 and Phase 7 was entered into analyses, and are described in detail in Supplementary Text S1.

### Assessment of nuisance variables

FSRS, MetS and AL index were included in a series of analyses, as either covariates of interest or no interest (nuisance variables). This was required to identify each marker’s unique association with brain structure, having controlled for the variance it shares with the other markers and socio-demographic variables. Furthermore, age at time of scan, sex, ethnicity, education and employment grade were used as nuisance variables and are described in detail in Supplementary Text S1.

### MRI acquisition and analysis

T_1_-weighted, and diffusion-weighted MRI images were acquired at the Oxford Centre for Functional MRI of the Brain (FMRIB), Wellcome Centre for Integrative Neuroimaging using a 3T Siemens Magnetom Verio (Erlangen, Germany) scanner with a 32-channel receive head coil, and were pre-processed and analysed using FSL v.5.0 tools^38^ described as part of the Whitehall II imaging sub-study protocol^29^. Details on MRI acquisition and processing are provided in Supplementary Text S2.

### Statistical analysis

Voxelwise general linear model (GLM) was applied for the analysis of grey matter and diffusion tensor imaging (DTI) data using Randomise^39^, a permutation-based non-parametric statistical test, running 5000 permutations and correcting for multiple comparisons across space. The significance threshold was set at *p* < 0.05, using threshold-free cluster enhancement ((TFCE))^40^. Three types of imaging-based statistical tests were run (details in Supplementary Text S3). (1) Simple linear t-tests of each marker in isolation, controlling for socio-demographics as nuisance variables:

$$\begin{array}{rcl}{\rm{Y}} & = & {\rm{\beta }}1\,{\rm{FSRS}}+{\rm{\beta }}2\,{\rm{age}}+{\rm{\beta }}3\,{\rm{sex}}+{\rm{\beta }}4\,{\rm{ethnicity}}+{\rm{\beta }}5\,{\rm{education}}+{\rm{\beta }}6\,{\rm{employment}}+{\rm{\varepsilon }}\\  &  & 1,\,0,\,0,\,0,\,0,\,0\\  &  & -1,\,0,\,0,\,0,\,0,\,0\\ {\rm{Y}} & = & {\rm{\beta }}1\,{\rm{MetS}}+{\rm{\beta }}2\,{\rm{age}}+{\rm{\beta }}3\,{\rm{sex}}+{\rm{\beta }}4\,{\rm{ethnicity}}+{\rm{\beta }}5\,{\rm{education}}+{\rm{\beta }}6\,{\rm{employment}}+{\rm{\varepsilon }}\\  &  & 1,\,0,\,0,\,0,\,0,\,0\\  &  & -1,\,0,\,0,\,0,\,0,\,0\\ {\rm{Y}} & = & {\rm{\beta }}1\,{\rm{AL}}+{\rm{\beta }}2\,{\rm{age}}+{\rm{\beta }}3\,{\rm{sex}}+{\rm{\beta }}4\,{\rm{ethnicity}}+{\rm{\beta }}5\,{\rm{education}}+{\rm{\beta }}6\,{\rm{employment}}+{\rm{\varepsilon }}\\  &  & 1,\,0,\,0,\,0,\,0,\,0\\  &  & -1,\,0,\,0,\,0,\,0,\,0\end{array}$$

(2) F-tests of pairs of markers, controlling for the third marker and socio-demographics as nuisance variables in order to determine the relative importance of specific markers on brain structure. Whenever a significant F-test was found, further post-hoc t-tests (3) were run to see if controlling for two of the three markers and socio-demographics as nuisance variables also yielded a result.

$$\begin{array}{rcl}{\rm{Y}} & = & {\rm{\beta }}1\,{\rm{FSRS}}+{\rm{\beta }}2\,{\rm{MetS}}+{\rm{\beta }}3\,{\rm{AL}}+{\rm{\beta }}4\,{\rm{age}}+{\rm{\beta }}5\,{\rm{sex}}+{\rm{\beta }}6\,{\rm{ethnicity}}+{\rm{\beta }}7\,{\rm{education}}\,+\\  &  & {\rm{\beta }}8\,{\rm{employment}}+{\rm{\varepsilon }}\\  &  & 0,-1,\,0,\,0,\,0,\,0,\,0,\,0,\,0\\  &  & 0,\,0,-1,\,0,\,0,\,0,\,0,\,0,\,0\\  &  & -1,\,0,\,0,\,0,\,0,\,0,\,0,\,0,\,0\\  &  & 0,0,-1,\,0,\,0,\,0,\,0,\,0,\,0\\  &  & -1,\,0,\,0,\,0,\,0,\,0,\,0,\,0,\,0\\  &  & 0,-1,\,0,\,0,\,0,\,0,\,0,\,0,\,0\end{array}$$

Results were localized using the Harvard-Oxford cortical and subcortical structural atlases for voxel-based morphometry (VBM) and the John Hopkins University DTI-based white matter atlases for tract-based special statistics (TBSS).

### Data availability

The study follows MRC data sharing policies [https://www.mrc.ac.uk/research/policies-and-guidance-for-researchers/data-sharing/]. Data will be accessible from the authors after 2019.

## Results

(Abbreviations used: FSRS: Framingham stroke risk score; MetS: metabolic syndrome; AL index: allostatic load index; GM: grey matter; DTI: diffusion tensor imaging; VBM: voxel-based morphometry; TBSS: tract-based spatial statistics)

### Descriptive statistics

#### Participant exclusion/inclusion

Participants with no T1 scan (N = 11), with structural abnormalities (N = 18) or inadequate quality T1 scan (N = 2), missing Framingham stroke risk (N = 25), metabolic syndrome (N = 4) or allostatic load score (N = 154) at either of the two phases, missing (N = 9) or un-useable DTI scan (N = 3) were excluded from analysis. VBM analysis was based on a final sample of N = 349 and TBSS on N = 337.

#### Socio-demographic variables

Mean follow-up time between Phase 3 and scan was 21.4 (SD 0.82) years. Participants were on average 69.6 (SD 5.2) years old. 80.5 percent in this study were male, with an average 14 years of education, which reflects the demographics of the British Civil Service in 1985 at recruitment to the Whitehall II study (Table 1). Marker characteristics used in the analysis are shown in Table 1 and Supplementary Table S2. Supplementary Table S2 summarizes their distribution in Phase 3 and Phase 7, and shows their increase across the two phases. Furthermore, 85% of participants were free from metabolic syndrome (MetS) and 38.7% of participants had an allostatic load (AL) index of less than 5, 50.2% between 5–9 and only 19.8% had an index from 10 to 15.

**Table 1:** Population and marker characteristics for Whitehall II imaging sub-study participants in Voxel-based morphometry (VBM) and Tract-based spatial statistics (TBSS) analyses.

	VBM (N = 349)	TBSS (N = 337)
**Socio-demographics**		
Age [years] *- Mean (SD), range*	69.6 (5.2), 60–83	69.5 (5.2), 60–83
Sex *- N (%) male*	281 (80.5)	271 (80.4)
Ethnicity - *N (%) white*	325 (93.1)	313 (92.9)
Occupation - *N (%)*		
Administrative (highest)	149 (42.7)	142 (42.1)
Professional/executive	171 (49.0)	167 (49.6)
Clerical/support (lowest)	29 (8.3)	28 (8.3)
Education level [years] *-Mean (SD), range*	13.9 (3.0), 6–23	14.0 (3.0), 6–23
**Marker characteristics**		
FSRS [%-risk of stroke in 10 years] *- Mean (SD), range*	8.5 (4.5), 2–42	8.4 (4.4), 2–42
Male - Mean (SD), range	9.47 (4.39), 6–42	9.34 (4.22), 6–42
Female - Mean (SD), range	4.51 (2.55), 2–13	4.56 (2.57), 2–13
MetS - *Mean (SD), range*	0.19 (0.47), 0–2	0.18 (0.45) 0–2
Male - Mean (SD), range	0.20 (0.50), 0–2	0.19 (0.47), 0–2
Female - Mean (SD), range	0.13 (0.34), 0–1	0.14 (0.35), 0–1
AL index – *Mean (SD), range*	5.65 (3.03), 0–15	5.62 (3.02), 0–15
Male - Mean (SD), range	5.86 (2.98), 0–15	5.84 (2.98), 0–15
Female - Mean (SD), range	4.75 (3.0), 0–11	4.73 (3.04), 0–11

The three markers were positively correlated. Participants with a high Framingham stroke risk (FSRS) had a high MetS score (Spearman’s rho (347) = 0.29, *p* < 0.001) and a high AL index (r_s_ (347) = 0.37, *p* < 0.001). Participants with a high MetS score also had a high AL index (r_s_ (347) = 0.53, *p* < 0.001). Males had significantly higher FSRS (t (347) = 8.93, *p* < 0.001) and AL index (t (347) = 2.75, *p* = 0.006) than females. MetS score was not significantly different between sexes (t (347) = 1.11, *p* = 0.27).

### Voxel-based morphometry results

#### Simple linear t-tests

**Figure 1:**
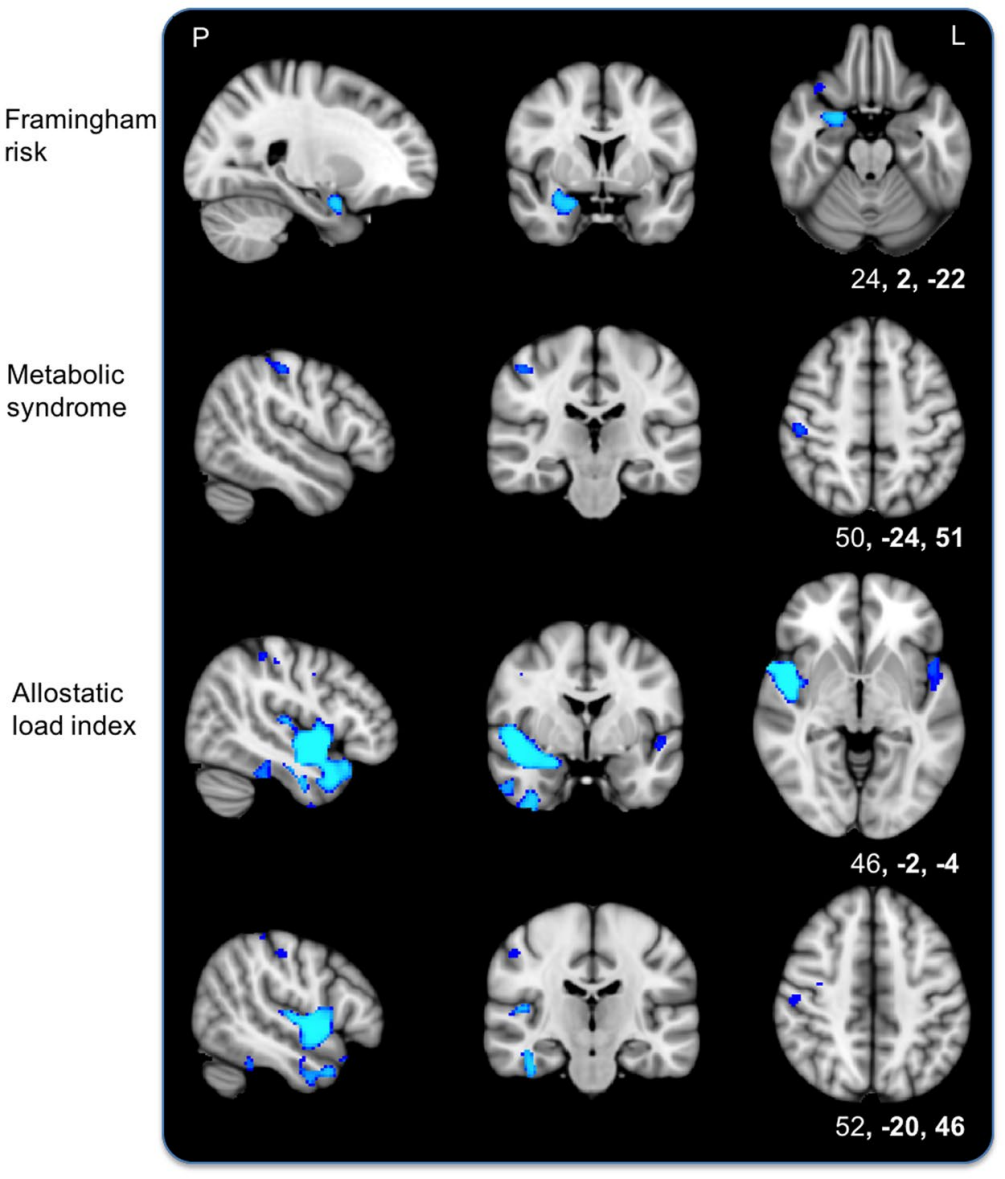
Simple linear t-tests of each marker and lower voxelwise grey matter density. T-tests show an association of higher Framingham stroke risk (top), metabolic syndrome (middle) and allostatic load index (bottom two rows) with lower voxelwise grey matter after removing the effects of socio-demographic variables. Blue represents regions significant at *p* < 0.05, threshold-free cluster enhancement, corrected for multiple comparisons. P, posterior; L, left. Coordinates are in MNI space.

After controlling for socio-demographic factors, voxelwise analyses of grey matter (GM) showed that higher FSRS, MetS and AL index were each separately associated with lower GM density. Significant voxels predominantly fell in the right hemisphere for each marker. For FSRS, results were primarily located within the right medial temporal lobe. For MetS, significant voxels were in the right postcentral gyrus. For AL index, significant regions included the right insular cortex, pre-, postcentral- and supramarginal gyrus, and the left central and frontal operculum (Fig. 1, Supplementary Table S3).

#### F-tests

**Figure 2:**
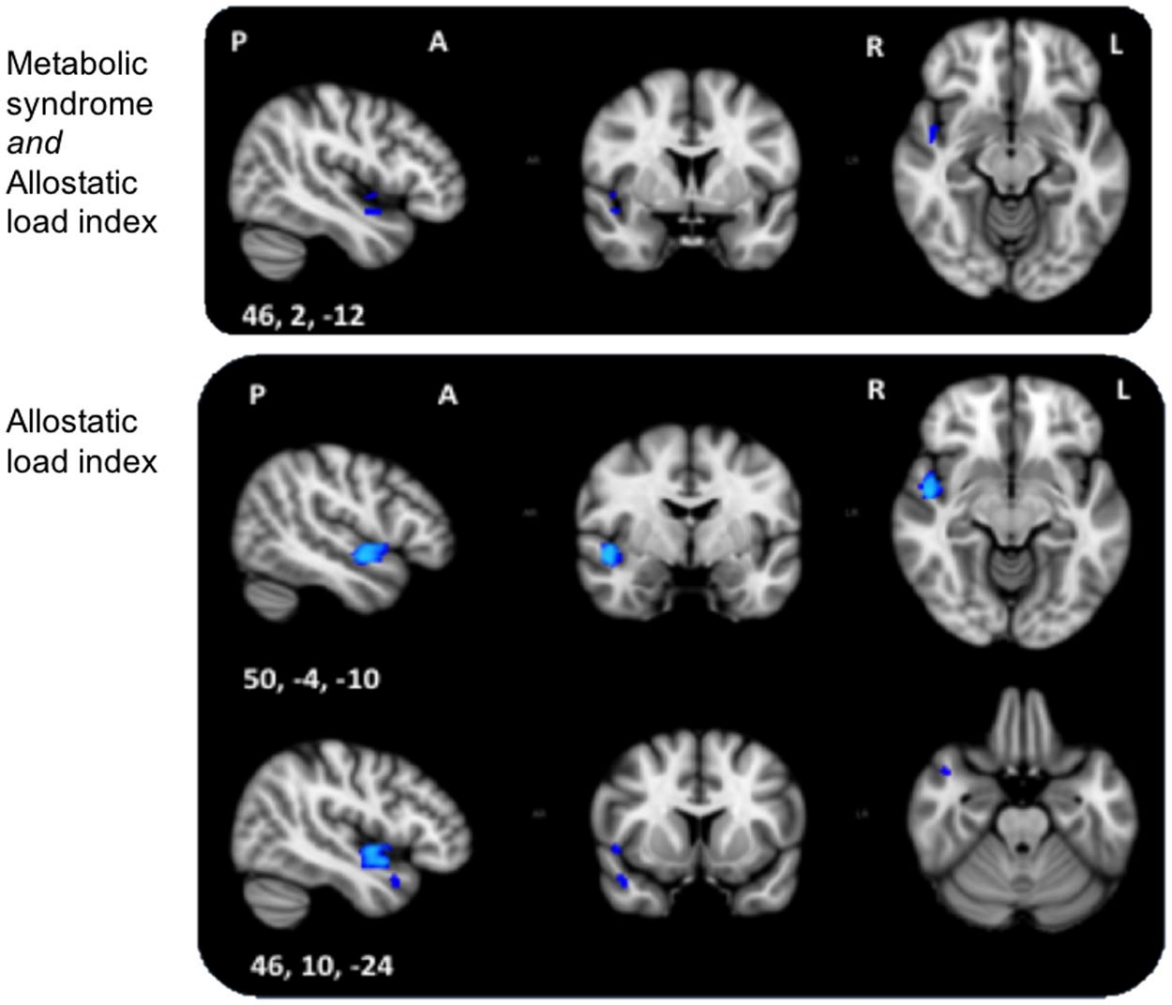
Top: F-test 1 of unique metabolic syndrome or allostatic load index association with grey matter after removing the effects of Framingham stroke risk and socio-demographic variables. Significant results extend the right insular and opercular cortex. Bottom: Post-hoc t-test shows an association between allostatic load index and lower voxelwise grey matter after removing the effects of metabolic syndrome, Framingham stroke risk and socio-demographic variables. Significant voxels were located along the right insular and opercular cortex, superior temporal gyrus and temporal pole. Blue represents regions significant at *p* < 0.05, threshold-free cluster enhancement, multiple comparisons corrected. P, posterior; A, anterior; R, right; L, left; Coordinates are in MNI space.

Only one of three F-tests showed significant associations with voxelwise GM. Significant associations of GM density with MetS or AL index or both extended to the right insular cortex, parts of the planum polare and Heschl’s gyrus in the opercular cortex. Maximum F-statistics were located in the right insular cortex (Fig. 2, Supplementary Table S4).

#### Post-hoc t-tests

Post-hoc t-tests showed a significant association between AL index and lower GM density after controlling for MetS, FSRS, and socio-demographic variables. Significant voxels were located in the right hemisphere in regions along the insular cortex, opercular cortex (planum polare, Heschl’s gyrus), superior temporal gyrus, and temporal pole (Fig. 2, Supplementary Table S4). No association of higher GM density was found with AL index, and no significant GM association was present with MetS. In summary, the markers could be ranked in terms of unique contribution to GM density as follows: AL > MetS > FSRS.

### Tract-based spatial statistics results: Fractional Anisotropy (FA)

#### Simple linear t-tests

**Figure 3:**
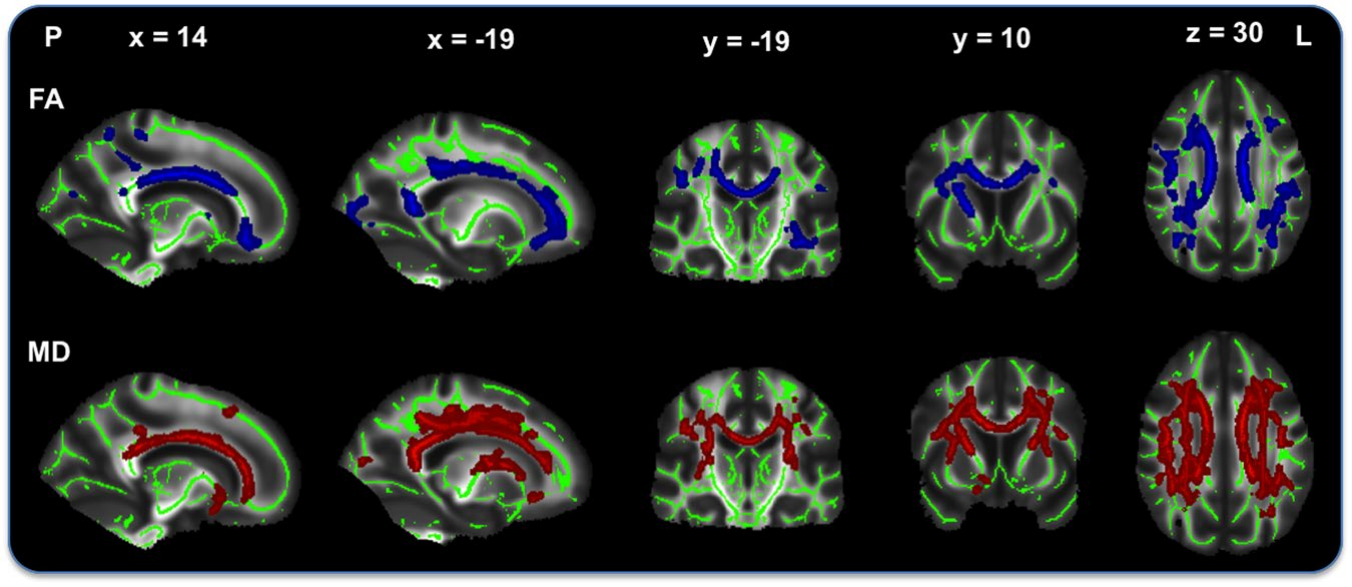
Simple linear t-test of Framingham stroke risk and lower white matter integrity. T-tests show an association of higher Framingham stroke risk with lower fractional anisotropy (FA; top) and higher mean diffusivity (MD; bottom) after removing the effects of socio-demographic variables. Results represent voxels significant at *p* < 0.05, threshold-free cluster enhancement, multiple comparisons corrected. Significant regions are dilated for illustrative purposes, overlaid on a green skeleton. P, posterior; L, left. Coordinates are in MNI space.

After controlling for socio-demographic factors, voxelwise analyses of fractional anisotropy (FA) showed that higher FSRS was associated with lower FA. Significant voxels were located bilaterally in the corona radiata (anterior, superior and posterior), corpus callosum (genu, body and splenium), inferior fronto-occipital fasciculus, inferior longitudinal fasciculus, forceps major, superior longitudinal fasciculus, in the right hemisphere along the anterior thalamic radiation, corticospinal tract and internal capsule, as well as in the left posterior thalamic radiation. Maximum t-statistic was located in the body of corpus callosum (Fig. 3, Supplementary Table S4). There was no positive association of higher FA and FSRS values, nor were there any associations between FA and MetS or AL index.

#### F-tests

**Figure 4:**
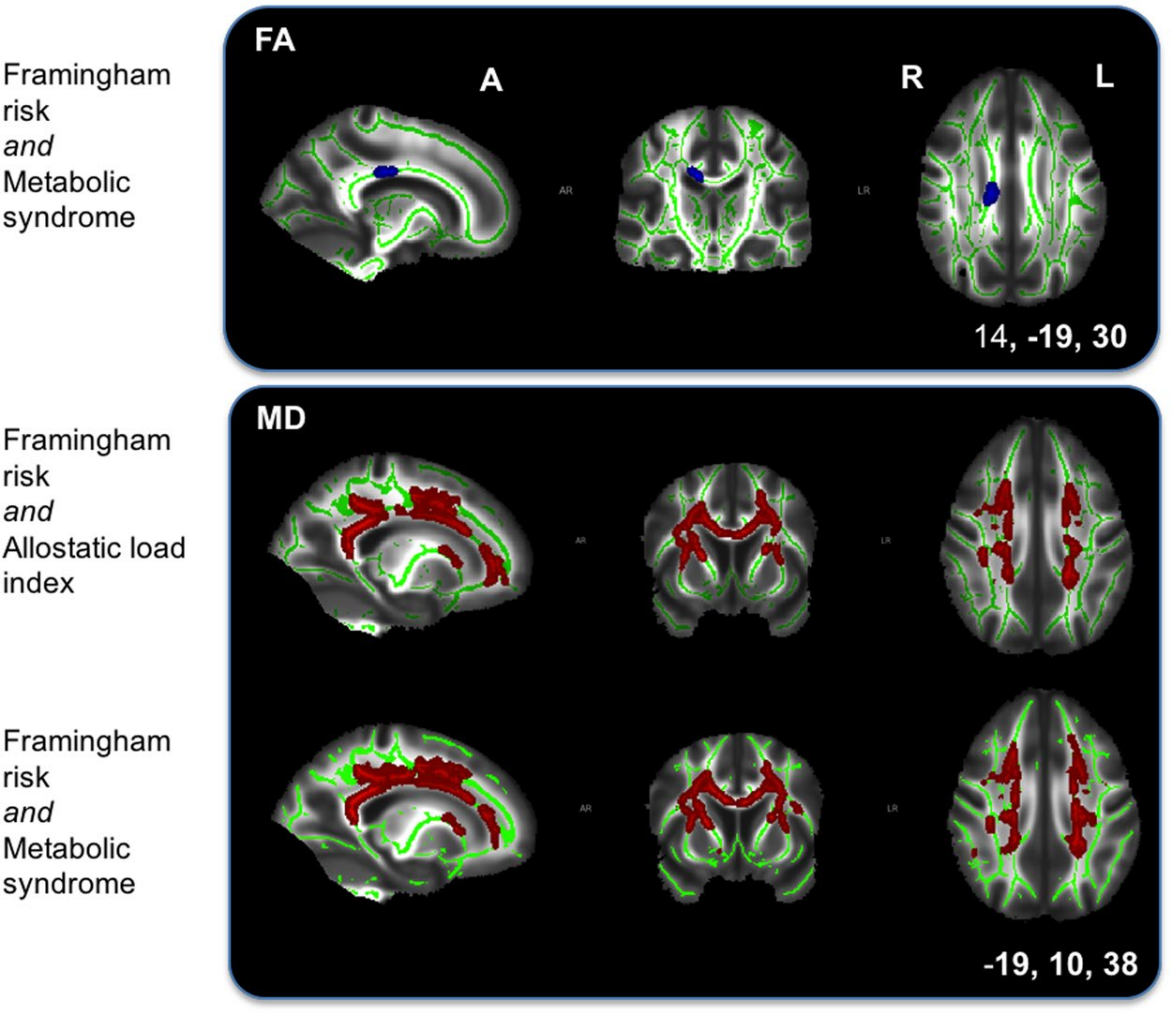
Top: F-test 3 of unique Framingham stroke risk or metabolic syndrome association with fractional anisotropy (FA) after removing the effects of allostatic load index and socio-demographic variables. Significant voxels are located in the body of corpus callosum. Bottom: F-test 2 (top) of unique Framingham stroke risk or allostatic load index and F-test 3 (bottom) of unique Framingham stroke risk or metabolic syndrome association with widespread mean diffusivity (MD) values. Results primarily extend the anterior thalamic radiation, corona radiata, corpus callosum, and internal and external capsule. Results represent voxels significant at *p* < 0.05, threshold-free cluster enhancement, multiple comparisons corrected. Significant regions are dilated for illustrative purposes, overlaid on a green skeleton and corrected for multiple comparisons. A, anterior; R, right; L, left. Coordinates are in MNI space.

Only one of three F-tests showed significant associations with voxelwise FA. The significant unique associations of FA values with FSRS or MetS or both were localized in the body of corpus callosum, after controlling for AL index and socio-demographics as nuisance variables (Fig. 4, Supplementary Table S4).

#### Post-hoc t-tests

**Figure 5:**
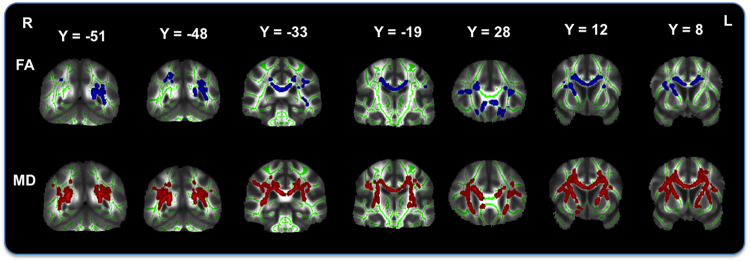
Post-hoc t-tests of Framingham stroke risk and lower white matter integrity. Post-hoc t-tests show an associations of higher Framingham stroke risk with lower fractional anisotropy (FA; top) and higher mean diffusivity (MD; bottom) after removing the effects of metabolic syndrome, allostatic load index and socio-demographic variables. Significant fractional anisotropy voxels extend bilaterally in the corona radiata, corpus callosum, forceps major, superior longitudinal fasciculus, in the right hemisphere along the anterior thalamic radiation, corticospinal tract and internal capsule, as well as in the left inferior fronto-occipital fasciculus and posterior thalamic radiation. Significant mean diffusivity voxels are present in similar regions but more widespread and less lateralised than with fractional anisotropy. Results represent voxels significant at *p* < 0.05, threshold-free cluster enhancement, multiple comparisons corrected. Significant regions are dilated for illustrative purposes, overlaid on a green skeleton. R, right; L, left. Coordinates are in MNI space.

Post-hoc t-tests showed a significant association between higher FSRS and lower FA values after controlling for MetS, AL index, and socio-demographic variables. Significant voxels were located in regions found in association with FSRS after removing socio-demographic factors (section 3.4.1, Fig. 3 and Supplementary Table S3) but their number was markedly reduced in the inferior fronto-occipital fasciculus bilaterally, in the right anterior thalamic radiation and corticospinal tract, and were no longer present in the right inferior and superior longitudinal fasciculus, and right half of the forceps major (Fig. 5, Supplementary Table S4) compared with the t-test of FSRS above. There was no positive association of FA with FSRS, nor any FA associations with MetS. In summary, the markers could be ranked in terms of unique contribution to white matter FA as follows: FSRS > MetS > AL.

### Tract-based spatial statistics results: Mean Diffusivity (MD)

#### Simple linear t-tests

After controlling for socio-demographic factors voxelwise analyses of mean diffusivity (MD) showed that higher FSRS was associated with higher MD. Significant voxels extended regions seen in association with FA (section 3.4.1) and were more widespread. Maximum t-statistic was located in the left anterior thalamic radiation (Fig. 3, Supplementary Table S3). No association of higher MD values was found with FSRS, and no significant MD association was present with MetS and AL index.

#### F-tests

Two out of three F-tests showed significant unique associations with voxelwise MD. Both F-tests revealed widespread significant unique associations of MD values with FSRS or AL index or both as well as FSRS or MetS or both after removing the effects of socio-demographic variables. Both F-tests revealed findings bilaterally in the anterior thalamic radiation, corona radiata (anterior, superior and posterior), corpus callosum (body, genu and splenium), internal capsule, external capsule and forceps major. Associations with MD values were revealed in the right tract of the superior longitudinal fasciculus in the former, and bilaterally in the latter F-test. Maximum F-statistics were located in the left anterior thalamic radiation for both tests (Fig. 4, Supplementary Table S4).

#### Post-hoc t-tests

After controlling for MetS, AL index and socio-demographic factors, a significant widespread association between higher FSRS and higher MD values was present in regions seen in association with FSRS after removing socio-demographic variables. Voxels were more widespread in the forceps minor and major, frontal part of the inferior fronto-occipital fasciculus, and the left anterior corona radiata before controlling for the other secondary stress markers (section 3.5.1). After additionally controlling for the markers, results were slightly more widespread in the anterior thalamic radiata and posterior part of the fronto-occipital fasciculus. Maximum t-statistics were located in the left anterior thalamic radiation (Fig. 5, Supplementary Table S4). Lower MD values were not associated with FSRS, and no significant MD association was present with AL index or MetS. In summary, the markers could be ranked in terms of unique contribution to white matter MD as follows: FSRS > AL, MetS.

## Discussion

We report findings from the first voxelwise study comparing three composite markers that have commonly been linked to stressors, health, and stress- and age-related disease. Hypothesis (1) was partially supported, i.e. Framingham stroke risk, metabolic syndrome and the allostatic load index were associated with lower grey matter density. Only Framingham stroke risk was associated with widespread reduced white matter integrity, namely lower fractional anisotropy and higher mean diffusivity values.

The markers had a unique association with structural brain integrity in older age after controlling for the effects of the other markers, and nuisance factors. However, the markers with the largest unique contribution were different for grey and white matter, respectively. A unique association of the allostatic load index measured across two study phases was found with lower grey matter in the right hemisphere in regions along the insular cortex, opercular cortex, superior temporal gyrus, and temporal pole. A unique association between FSRS and widespread lower white matter was also found.

### Grey matter (possible mechanisms)

The allostatic load index was the best candidate marker to affect brain cortex, followed by the metabolic syndrome then Framingham stroke risk. It uniquely accounts for risk that is not related to vascular risk captured by the Framingham stroke risk or metabolic syndrome. A single study supports concurrently measured allostatic load in predicting MRI brain structure in older age^19^. In our study, the allostatic load index did not affect the brain structures typically predicted, such as the hippocampal formation and prefrontal cortex^41^. The location of effects in lateral temporo-frontal regions may coincide with the distribution of the middle cerebral artery. Infarcts are most common in this vascular territory^42–44^, so this may point to a vascular nature of the underlying mechanisms. Although there is great variability in how the allostatic load index is derived, it is often calculated as the sum of the largest number of markers above respective thresholds^9^. This results in a large coefficient of variance making it more likely that an association is found. Although the Framingham stroke risk score had the largest variance, it is also closely associated with age, which is a strong predictor of grey matter atrophy^45^, and if controlled for, removes the shared variance between the Framingham stroke risk score and grey matter.

The allostatic load index was the least associated with confounding variables, such as age, as largest associations with grey matter were found with allostatic load index after removing the effect of socio-demographic variables. Unlike in the case of the Framingham stroke risk score, age is not incorporated in the index^9^. Allostatic load is conceptualized as the accumulation of stress responses over time, thus it theoretically represents the accumulated damage of the allostatic process on the body over the life course^46^. Although it is often assumed that allostatic load increases with the passage of time even if it was low in early adulthood^19^, a cross-sectional study found that, whereas allostatic load increased from the 20s into the 60s, levels of the index stabilized in the 70s and 80s^47^. This could be a reflection of those with lower allostatic load index continuing to live into older age. To date only a few prospective studies support the applicability of the allostatic load concept empirically^13,28,48,49^. Therefore, prospective studies that longitudinally and concurrently assess allostatic load and brain structure are needed in order to identify critical periods where the ageing brain is particularly sensitive to allostatic load, as well as studies that examine the structural brain markers of allostatic change from early adulthood to mid-life^50^.

### White matter (possible mechanism)

The Framingham stroke risk score was the best candidate marker to predict white matter integrity, followed by metabolic syndrome and then the allostatic load index for fractional anisotropy, while for mean diffusivity it was metabolic syndrome or allostatic load index to an equal extent.

#### Vascular risk and stroke

The Framingham stroke risk score provides a clinically validated stroke risk profile based on the history and presence of cardiovascular risk factors, which in the present study was also associated with white matter structure. Vascular risk has been acknowledged to contribute widely to white matter changes appearing hyperintense on FLAIR images^51–53^ and in relation to DTI measures^54^. White matter changes have been linked to the pathology of stroke^55,56^, dementia^57–59^, and risk of death^60^. Around 25% of all strokes are accounted for by small vessel infarcts of the white matter^27,61^. Cardiovascular risk factors such as type II diabetes are associated with small vessel disease^62^.

#### Vascular risk and dementia

Over time, brain-regulated sympathetic autonomic nervous system activity leads to the wear-and-tear of the cardiovascular system, which in turn affects brain regional vasodilation and vascular reactivity that are required for tasks and clearing of waste products. These in turn increase the risk of dementia^63^.

The link between vascular factors and dementia is well known^64^. Vascular factors manifest as white matter lesions and lacunes, and increase the clinical expression of dementia at a certain burden of Alzheimer pathology^57,58,65^. Hypertension, smoking and hypercholesterolemia, which are part of the Framingham stroke risk score, are clinical risk factors for the clinical diagnosis of Alzheimer’s disease and for the presence of Alzheimer pathology^66^. Alzheimer pathology in the form of cerebral amyloidosis can affect vascular and endothelial function, which in turn might lead to impaired vascular mechanisms and clearing of abnormal proteins, such as amyloid, from the brain^65,67^.

### Strengths and limitations

Study strengths are the relatively large sample size, repeated measures of markers, and up-to-date imaging techniques. Limitations are the cross-sectional imaging design, the underrepresentation of females in this study sample and the lack of primary marker components in the allostatic load index. This sex imbalance is representative of the Whitehall II cohort at baseline^68^, and at the recruitment phase (Phase 11). Sex differences in age trajectories of certain physiological disorders, such as cardiovascular and central obesity, are reflected in and accounted for in the Framingham and metabolic syndrome scores^69^. While it would be possible to refine risk, e.g. allostatic load, by using sex-specific cut-offs, we limited our approach to using published, generally accepted definitions. The generation of a composite best predictor index would be possible given our data, but was outside the scope of this paper. Sex differences in subjective stress perception and multi-factorial physiological dysregulation through the lifespan have also been reported^70^. However, due to a relatively higher vascular mortality rate amongst women that coincides with female reproductive decline, the sex gap in ill-health and mortality is reduced in postmenopausal age^71^. In addition, in the present analysis we used the sum of composite markers from two study phases, twenty and ten years prior to the scan, which reduced any statistical variability due to markers acquired pre-menopause. Components of the allostatic load index were also limited to secondary markers of the stress response, which might explain why the allostatic load index was not associated with brain regions that regulate the stress response, namely the prefrontal cortex, hippocampus, amygdala or hypothalamus.

Sex-balanced prospective longitudinal cohorts that concurrently measure changes in allostatic load markers and brain structure will help us understand the sex differences in age trajectories of physiological dysregulation and structural brain changes. In turn these can serve as risk factors for tertiary outcomes of allostatic overload, such as Alzheimer’s disease and mortality^9^.

### Future directions

Prospective cohort studies that longitudinally and concurrently assess stressors, the allostatic load index and brain structure are needed in order to tease apart the allostasis and allostatic load mechanisms, which are involved in ageing. Recent research focused on identifying the subtly abnormal patterns of brain ageing that precede cognitive decline and the development of Alzheimer’s pathology^72^. Mechanisms in which composite stress markers come together to predict brain changes^73^, cognitive decline^74^, and what role gender plays in these^75^, needs further clarification^50^. Understanding the process that links allostatic load mechanisms to health outcomes and their multifactorial predictors, as well as disease trajectories before the illness develops, will benefit research into age-related and neurodegenerative diseases.

## Electronic supplementary material

Supplement
