# Supplementary material for: Allostatic load as a predictor of grey matter volume and white matter integrity in old age: The Whitehall II MRI study
Source: Sci Rep. 2018 Apr 23;8:6411. doi: 10.1038/s41598-018-24398-9 (PMC5913085; doi:10.1038/s41598-018-24398-9)
Supplement: Supplementary file 1 — Supplement [file 41598_2018_24398_MOESM1_ESM.docx]

**Allostatic load as a predictor of grey matter volume and white matter integrity in old age: The Whitehall II MRI study**

Enikő Zsoldos*^1,2^, DPhil, Nicola Filippini^1,2^, DPhil, Abda Mahmood^1^, MSc, Clare E Mackay^1,3^, PhD, Archana Singh-Manoux^4,5^, PhD, Mika Kivimäki^4^, PhD, Mark Jenkinson^2^, DPhil, Klaus P Ebmeier^1^, MD

^1^Department of Psychiatry, University of Oxford, Warneford Hospital, Oxford, OX3 7JX, UK

^2^Wellcome Centre for Integrative Neuroimaging, Oxford Centre for Functional MRI of the Brain, Nuffield Department of Clinical Neurosciences, University of Oxford, John Radcliffe Hospital, Oxford, OX3 9DU, UK

^3^Wellcome Centre for Integrative Neuroimaging, Oxford Centre for Human Brain Activity, University of Oxford, Warneford Hospital, Oxford, OX3 7JX, UK

^4^Department of Epidemiology and Public Health, University College London, London, WC1E 7HB, UK

^5^Centre for Research in Epidemiology and Population Health, INSERM, U1018, France

*Corresponding Author: Dr Enikő Zsoldos, Department of Psychiatry, University of Oxford, Warneford Hospital, Oxford, OX3 7JX, UK, e-mail: eniko.zsoldos@psych.ox.ac.uk

Supplementary Text S1. Assessment of markers and nuisance variables

**Framingham stroke risk score**

The Framingham stroke risk score (FSRS) is a sex-specific stroke risk appraisal function that empirically relates cardiovascular risk factors to the probability of a stroke within 10 years.^1^ Risk factors include cardiovascular health (systolic blood pressure, prior cardiovascular disease, atrial fibrillation, left ventricular hypertrophy, antihypertensive medication), diabetes mellitus, smoking habits, sex and age. The percentage risk score was computed using beta coefficients based on the Cox proportional hazards regression model in the Framingham study. The sum of risk at Phase 3 and Phase 7 was entered into analyses.

**Metabolic syndrome**

Metabolic syndrome (MetS) was defined using the National Cholesterol Education Program criteria,^2^ based on the presence of at least three of the following five components: blood pressure: ≥ 130/85 mmHg or use of antihypertensive medication, waist circumference (abdominal obesity): men > 102 cm, women > 88 cm, fasting glucose: ≥ 6.11 mmol/L, HDL cholesterol: men < 1.03 mmol/L, women < 1.30 mmol/L, and serum triglycerides: ≥ 1.70 mmol/L (Table 1). A binarized (yes/no) score was given if the criteria were met at a single phase before summing the binary scores of the two phases.

**Allostatic load index**

Allostatic load (AL) index was defined as the linear combination of nine physiological measures with values above a high-risk threshold.^3^ Following standard practice, the 75th percentile distribution-based cut-off was used, where clinically relevant cut-off norms have not been established (here: fasting insulin and IL-6).^4^ AL index was defined at phases 3 and 7 based on the following components and their cut-offs: blood pressure: ≥ 140/90 mmHg, BMI: ≥ 25 kg/mm², fasting glucose: ≥ 5.50 mmol/L, fasting insulin: ≥ 7.53 mcU/ml, high-density (HDL) lipoprotein cholesterol: < 1.03 mmol/L, low-density lipoprotein (LDL) cholesterol: ≥ 3.36 mmol/L, serum triglycerides: ≥ 1.70 mmol/L, C-reactive protein (CRP): and interleukin-6 (IL-6): ≥ 1.85 pg/ml (Supplementary Table 1). An elevated level of each measure carries more risk, except in case of HDL cholesterol. The same cut-off values were used for both phases and distribution-based cut-offs were established based on Phase 3 values. Each component was given the same weighting and was dichotomized (yes/no) based on its respective threshold. The dichotomized scores were summarized to form an AL index at each phase.

**Assessment of nuisance variables**

**Markers**

Framingham stroke risk score (FSRS), metabolic syndrome (MetS) and allostatic load (AL) index were included in a series of analyses, variably as either covariates of interest or no interest (nuisance variables). This was required to identify each marker’s unique association with brain structure, having controlled for the variance it shares with the other markers and socio-demographic variables.

**Socio-demographics**

Age at time of scan, sex, ethnicity, education and employment grade were used as nuisance variables. Ethnicity was defined as white and non-white. Education years were calculated as the difference between the age at which the participant commenced primary school and the age at which they first left full-time education. Socioeconomic status was classified according to occupation grade at Phase 1: senior managers and administrators (highest grade), professionals and executives (middle grade), and clerical and support staff (lowest grade).

Supplementary Text S2. MRI acquisition and processing

**Grey matter density**

Voxelwise analysis of grey matter (GM) was performed using FSL-VBM^5^ an optimised voxel-based morphometry (VBM) protocol in the FMRIB Software Library (FSL) version 5.0.^6^ Normalized bias-corrected brain extracted images were grey matter segmented before being registered to the Montreal Neurological Institute (MNI) 152 standard space using FMRIB’s non-linear registration tool (FNIRT^7^). The images were averaged and flipped along the x-axis to create a left-right symmetric, study-specific grey matter template. All native grey matter images were non-linearly registered to this study-specific template and modulated to correct for local expansion (or contraction) due to the non-linear component of the spatial transformation. The modulated grey matter images were then smoothed with an isotropic Gaussian kernel with a sigma of 3 mm. Finally, a study-specific grey matter mask was used to enable accurate localization of results in an ageing sample.

**White matter microstructure**

In diffusion tensor imaging (DTI) analysis, images were corrected for susceptibility-induced distortions using two b=0 scans (b-value 0 s/mm^2^), acquired with opposing phase-encoding directions using the FSL topup tool.^8^ Eddy current-induced distortions and subject movement were corrected using the FSL tool eddy.^9^ This employs a second order polynomial model and transforms each scan towards the Gaussian process predicted scan. It also identifies outlier slices (dropout) caused by movement during diffusion encoding.^10^ Slices were classified as outliers and replaced if the signal was found to be more than 3 SD from the Gaussian Process predicted slice. The volume was removed if over 10 slices were identified as outliers within a volume. The scan was excluded from analysis if more than five volumes were removed.

Voxelwise statistical analysis of fractional anisotropy (FA) and mean diffusivity (MD) was carried out using tract-based spatial statistics ((TBSS)^11^). A tensor model was fitted to the raw diffusion data using DTIFit part of FMRIB’s Diffusion Toolbox (<http://fsl.fmrib.oc.ac.uk/fsl/fdt>) to create FA maps. This fits a diffusion tensor model to the raw diffusion data and then brain-extracts using BET.^12^ All subjects' FA data were then aligned into a common space using FNIRT,^7^ which uses a b-spline representation of the registration warp field.^13^ Next, the mean FA image was created and thinned to create a mean FA skeleton, which represents the centers of all tracts common to the group. Each subject's aligned FA data were then projected onto this skeleton and the resulting data fed into voxelwise cross-subject statistics. This method was repeated for MD.

Supplementary Text S3. Statistical analysis

(Abbreviations used: FSRS: Framingham stroke risk score; MetS: metabolic syndrome; AL index: allostatic load index; GM: grey matter; DTI: diffusion tensor imaging)

Three types of imaging-based statistical tests were run. (1) simple linear t-tests of each marker in isolation, controlling for socio-demographics as nuisance variables:

Y = β_1_ FSRS + β_2_ age + β_3_ sex + β_4_ ethnicity + β_5_ education + β_6_ employment + ε

1, 0, 0, 0, 0, 0

-1, 0, 0, 0, 0, 0

Y = β_1_ MetS + β_2_ age + β_3_ sex + β_4_ ethnicity + β_5_ education + β_6_ employment + ε

1, 0, 0, 0, 0, 0

-1, 0, 0, 0, 0, 0

Y = β_1_ AL + β_2_ age + β_3_ sex + β_4_ ethnicity + β_5_ education + β_6_ employment + ε

1, 0, 0, 0, 0, 0

-1, 0, 0, 0, 0, 0

(2) F-tests of pairs of markers, controlling for the third marker and socio-demographics as nuisance variables; (3) post-hoc t-tests based on the F-test results, controlling for the other two markers and socio-demographics as nuisance variables. The nature of the F-test is to ask a question (or formulate a hypothesis) along the lines of: is the FSRS or MetS or AL index effect, or any combination of them, significantly non-zero? More specifically, the following three F-tests were run in order to determine the relative importance of specific markers on brain structure: F-test 1 of MetS and AL index controlling for FSRS and socio-demographics as nuisance variables; F-test 2 of FSRS and AL index controlling for MetS and socio-demographics as nuisance variables; and F-test 3 of FSRS and MetS controlling for AL index and socio-demographics as nuisance variables. Whenever a significant F-test was found, further post-hoc t-tests were run to see if controlling for two of the three markers and socio-demographics as nuisance variables also yielded a result. For example, F-test 1 results indicate whether MetS or AL index or both are correlated with voxelwise GM and DTI data, after controlling for FSRS and socio-demographics as nuisance variables. If F-test 1 was significant, a post-hoc t-test of MetS (t-test 1, t1) and AL index (t-test 2, t2) with voxelwise GM and DTI measures were run, controlling for the other markers and socio-demographics as nuisance variables. If F-test 2 was significant, a post-hoc t-test of FSRS (t-test 3, i.e. t3) and AL index (t-test 4, i.e. t4) were run, controlling for the other markers and socio-demographics as nuisance variables. Finally, if F-test 3 was significant, a post-hoc t-test of FSRS (t-test 5, i.e. t5) and MetS (t-test 6, i.e. t6) were run, controlling for the other markers and socio-demographics as nuisance variables.

Y = β_1_ FSRS + β_2_ MetS + β_3_ AL + β_4_ age + β_5_ sex + β_6_ ethnicity + β_7_ education + β_8_ employment + ε

0, -1, 0, 0, 0, 0, 0, 0, 0

0, 0, -1, 0, 0, 0, 0, 0, 0

-1, 0, 0, 0, 0, 0, 0, 0, 0

0, 0, -1, 0, 0, 0, 0, 0, 0

-1, 0, 0, 0, 0, 0, 0, 0, 0

0, -1, 0, 0, 0, 0, 0, 0, 0

Supplementary Table S1. Metabolic syndrome and allostatic load index composite measure cut-offs.

|  | Metabolic Syndrome | Allostatic  Load Index |
| --- | --- | --- |
| Blood pressure | ≥ 130/85 mmHg | ≥ 140/90 mmHg |
|  | Antihypertensives |  |
| Waist circumference |  | N/A |
| Men | > 102cm |  |
| Women | > 88cm |  |
| Fasting glucose | ≥ 6.11 mmol/L | ≥ 5.50 mmol/L |
| HDL cholesterol |  |  |
| Men | < 1.03 mmol/L | < 1.03 mmol/L |
| Women | < 1.30 mmol/L |  |
| Serum triglycerides | ≥ 1.70 mmol/L | ≥ 1.70 mmol/L |
| Body mass index | N/A | ≥ 25 kg/mm² |
| Fasting insulin | N/A | ≥ 7.53 mcU/ml |
| LDL cholesterol | N/A | ≥ 3.36 mmol/L |
| CRP | N/A | ≥ 3.00 mg/L |
| IL-6 | N/A | ≥ 1.85 pg/ml |

Comparison of the metabolic syndrome and allostatic load ndex composite measure cut-offs. An elevated level of each measure carries more risk, except in case of HDL cholesterol. LDL, Low-density lipoprotein; HDL, High-density lipoprotein; CRP, C-reactive protein; IL-6, Interleukine-6.

Supplementary Table S2. Average (SD) Framingham stroke risk and allostatic load indices at Phase 3 and Phase 7, as well as binary frequency [%, yes] and 95% exact binomial confidence intervals (CI) of metabolic syndrome not directly used for analysis. Total scores across both phases used in voxelwise grey matter analysis are in Table 1.

|  | Phase 3 1991-1993 | Phase 7 2003-2004 |
| --- | --- | --- |
| Age [years] – *Mean (SD)* | 48.2 (5.2) | 59.4 (5.2) |
| Framingham 10-year Stroke Risk [%] - *Mean (SD)* | 3.3 (1.4) | 5.2 (3.7) |
| Metabolic Syndrome *– % yes (95% CI)* | 8.0 (5.4 – 11.3) | 10.9 (7.8 – 14.6) |
| Allostatic Load *- Mean (SD)* | 2.7 (1.7) | 3.0 (1.8) |

Supplementary Table S3**.** Significant clusters of voxelwise grey matter and fractional anisotropy (FA) negatively associated with each marker. Significant clusters of voxelwise mean diffusivity (MD) positively associated with Framingham stroke risk.

|  |  | **No of voxels** | **Max t** | ***p*-value** | **MNI coordinates  (x, y, z)** | | | | **Structure** | |
| --- | --- | --- | --- | --- | --- | --- | --- | --- | --- | --- |
| Grey matter (GM) | Framingham risk | 194 | 5.09 | 0.008 | 24 | 2 | -22 | R amygdala, parahippocampal gyrus anterior division | |  |
|  |  | 21 | 4.31 | 0.042 | 36 | 20 | -20 | R frontal orbital cortex,  temporal pole | |  |
|  |  | 14 | 4.52 | 0.045 | -44 | -20 | 10 | L Heschl's Gyrus (includes H1 and H2), central opercular cortex | |  |
|  | Metabolic syndrome | 69 | 4.70 | 0.028 | 48 | -24 | 50 | R postcentral gyrus | |  |
|  | Allostatic load | 3413 | 5.21 | < 0.001 | 46 | -2 | -4 | R planum polare,  insular cortex | |  |
|  |  | 134 | 3.99 | 0.033 | -48 | 8 | -4 | L central opercular cortex, frontal operculum cortex | |  |
|  |  | 44 | 3.40 | 0.045 | 40 | -10 | 40 | R precentral gyrus | |  |
|  |  | 35 | 3.40 | 0.046 | 52 | -20 | 46 | R postcentral gyrus | |  |
|  |  | 32 | 3.87 | 0.038 | 48 | -34 | 54 | R supramarginal gyrus anterior and posterior divisions | |  |
|  |  | 4 | 3.38 | 0.049 | 44 | 0 | 40 | R precentral gyrus | |  |
| FA | Framingham risk | 15710 | 4.97 | 0.012 | 14 | -19 | 30 | Body of corpus callosum | |  |
| MD | Framingham risk | 19664 | 7.63 | 0.006 | -19 | 10 | 38 | L anterior  thalamic radiation | |  |

Supplementary Table S4. Significant clusters of voxelwise grey matter (GM) negatively associated with allostatic load (AL) index. Framingham stroke risk score (FSRS) was negatively associated with clusters of fractional anisotropy (FA) and positively with mean diffusivity (MD).

|  |  | **No of voxels** | **Max statistic** | ***p*-value** | **MNI coordinates  (x, y, z)** | | | | **Structure** | |
| --- | --- | --- | --- | --- | --- | --- | --- | --- | --- | --- |
| Grey matter | MetS or AL  F (1,340) | 38 | 11.70 | 0.042 | 46 | 2 | -12 | R insular cortex | |  |
|  | AL index  t (340) | 280 | 4.61 | 0.009 | 50 | -4 | -10 | R planum polare | |  |
|  |  | 17 | 4.03 | 0.046 | 46 | 10 | -24 | R temporal pole | |  |
| Fractional anisotropy | FSRS or MetS F (1,329) | 67 | 11.40 | 0.045 | 14 | -19 | 30 | Body of  corpus callosum | |  |
|  | FSRS t (329) | 4110 | 4.64 | 0.020 | 14 | -19 | 30 | Body of  corpus callosum | |  |
|  |  | 1933 | 4.17 | 0.037 | -21 | -51 | 9 | Forceps major | |  |
|  |  | 1276 | 4.66 | 0.033 | 32 | 12 | 17 | R anterior  corona radiata | |  |
|  |  | 1142 | 4.40 | 0.042 | -29 | 28 | 14 | L inferior fronto-occipital fasciculus | |  |
|  |  | 65 | 2.92 | < 0.050 | 20 | 8 | 12 | R anterior  thalamic radiation | |  |
|  |  | 28 | 1.98 | < 0.050 | -34 | -33 | 25 | L superior  longitudinal fasciculus | |  |
|  |  | 21 | 2.02 | < 0.050 | -38 | -48 | 17 | L superior  longitudinal fasciculus | |  |
|  |  | 5 | 3.28 | < 0.050 | 16 | -53 | 56 | R anterior  thalamic radiation | |  |
| Mean diffusivity | FSRS or AL F (1,329) | 10760 | 24.60 | 0.013 | -19 | 10 | 38 | L anterior  thalamic radiation | |  |
|  | FSRS or MetS F (1,329) | 11550 | 28.8 | 0.008 | -19 | 10 | 38 | L anterior  thalamic radiation | |  |
|  |  | 152 | 7.28 | 0.048 | -30 | -26 | 4 | L internal capsule | |  |
|  |  | 101 | 10.40 | 0.047 | 37 | 15 | 18 | R superior  longitudinal fasciculus | |  |
|  | FSRS t (329) | 19561 | 7.01 | 0.007 | -19 | 10 | 38 | L anterior  thalamic radiation | |  |

**References**

1 D'Agostino, R. B., Wolf, P. A., Belanger, A. J. & Kannel, W. B. Stroke risk profile: adjustment for antihypertensive medication. The Framingham Study. *Stroke* **25**, 40-43 (1994).

2 Expert Panel on Detection Evaluation and Treatment of High Blood Cholesterol In Adults (Adult Treatment Panel III). Third Report of The National Cholesterol Education Program (NCEP) - Executive Summary. *JAMA* **285**, 2486-2497 (2001).

3 Dich, N., Lange, T., Head, J. & Rod, N. H. Work stress, caregiving, and allostatic load: prospective results from the Whitehall II cohort study. *Psychosom Med* **77**, 539-547, doi:10.1097/PSY.0000000000000191 (2015).

4 Juster, R. P., McEwen, B. S. & Lupien, S. J. Allostatic load biomarkers of chronic stress and impact on health and cognition. *Neurosci Biobehav Rev* **35**, 2-16, doi:10.1016/j.neubiorev.2009.10.002 (2010).

5 Douaud, G. *et al.* Anatomically related grey and white matter abnormalities in adolescent-onset schizophrenia. *Brain* **130**, 2375-2386, doi:10.1093/brain/awm184 (2007).

6 Good, C. D. *et al.* A voxel-based morphometric study of ageing in 465 normal adult human brains. *Neuroimage* **14**, 21-36, doi:10.1006/nimg.2001.0786 (2001).

7 Andersson, J., Jenkinson, M., Smith, S. *Non-linear registration, aka spatial normalisation. www.fmrib.ox.ac.uk/analysis/techrep/ 2007/TR07JA2*, 2010).

8 Andersson, J. L. R., Skare, S. & Ashburner, J. How to correct susceptibility distortions in spin-echo echo-planar images: application to diffusion tensor imaging. *Neuroimage* **20**, 870-888, doi:10.1016/S1053-8119(03)00336-7 (2003).

9 Andersson, J. L. R. & Sotiropoulos, S. N. An integrated approach to correction for off-resonance effects and subject movement in diffusion MR imaging. *Neuroimage* **125**, 1063-1078, doi:10.1016/j.neuroimage.2015.10.019 (2016).

10 Andersson, J. L., Graham, M. S., Zsoldos, E. & Sotiropoulos, S. N. Incorporating outlier detection and replacement into a non-parametric framework for movement and distortion correction of diffusion MR images. *Neuroimage*, doi:10.1016/j.neuroimage.2016.06.058 (2016).

11 Smith, S. M. *et al.* Tract-based spatial statistics: voxelwise analysis of multi-subject diffusion data. *Neuroimage* **31**, 1487-1505, doi:10.1016/j.neuroimage.2006.02.024 (2006).

12 Smith, S. M. Fast robust automated brain extraction. *Hum Brain Mapp* **17**, 143-155, doi:10.1002/hbm.10062 (2002).

13 Rueckert, D. *et al.* Nonrigid registration using free-form deformations: application to breast MR images. *IEEE Trans Med Imaging* **18**, 712-721, doi:10.1109/42.796284 (1999).
